# COVAX and the many meanings of sharing

**DOI:** 10.1136/bmjgh-2021-007763

**Published:** 2021-11-23

**Authors:** Katerini Tagmatarchi Storeng, Felix Stein, Antoine de Bengy Puyvallée

**Affiliations:** 1 Centre for Development and the Environment, University of Oslo, Oslo, Norway; 2 London School of Hygiene & Tropical Medicine, London, UK

**Keywords:** COVID-19, health policy, vaccines, global health, public-private partnerships

**Summary box**

- The COVID-19 Vaccines Global Access Facility (COVAX) has been promoted as the ‘only global solution’ to end the accute phase of the COVID-19 pandemic. The notion of ‘sharing’ has been a central trope in policy debate about this ‘vaccine-sharing’ scheme.
- In the first eighteen months of the pandemic, COVAX has struggled to share the risks and benefits of vaccine development, wealthy countries’ excess vaccine doses, and the burden of financing COVAX and the wider Access to COVID-19 Tools Accelerator (ACT-A), of which it is part.
- In the global pandemic response, the term ‘sharing’ glosses various kinds of transactions, some of which jar with commonplace understandings of what it means ‘to share’.
- COVAX’s approach to vaccine sharing obscures what it does *not* share: decision-making power and the knowledge and technology to produce vaccines everywhere.

## Introduction

The notion of ‘sharing’ has been a central trope in the global response to COVID-19. The slogan ‘no one is safe until everyone is safe’, which has been repeated ad nauseam during the pandemic, presupposes that COVID-19 is a threat to all of humanity, requiring collective solutions. Nowhere is this more evident than in the global ‘vaccine-sharing’ scheme COVAX (formally known as The COVID-19 Vaccines Global Access Facility), which was established to enable the rapid development and equitable distribution of COVID-19 vaccines globally. As Seth Berkley, Chief Executive Officer of Gavi, the Vaccine Alliance, which co-leads COVAX, argued in the early months of the pandemic: “While there are no guarantees that any Covid-19 vaccine candidates will ultimately succeed […] sharing the risks through the [COVAX] Facility offers our best shot at beating this virus by enabling the world to share the rewards.”^1^ But what has ‘sharing’ meant in practice during the first eighteen months of the pandemic?

In short, as a ‘vaccine-sharing’ scheme, COVAX offered to share three things: the risks and benefits of vaccine development, vaccine doses, and the burden of financing COVAX and Access to COVID-19 Tools Accelerator (ACT-A), of which it is part. In each of these instances, the term ‘sharing’ glosses various kinds of transactions, some of which jar with commonplace understandings of what it means ‘to share’ and serve to obscure what COVAX has not shared—notably decision-making power and the knowledge and technology to produce vaccines. Examining specific uses of the term ‘sharing’ is important because they shape moral and moralistic discourses that put pressure on different actors and establish who may have done their ‘fair share’ to help end the pandemic.

## Risk sharing

From its inception, COVAX argued that pandemic risk should be seen as an extension of the risks of corporate vaccine research and development (R&D).^2^ It underlined how lengthy, risky and expensive corporate vaccine development tended to be and that it should thus not be left to market forces alone.^1 3^ COVAX’s response was to use public funding from participating governments to ‘share’ corporate risk with pharmaceutical companies through various R&D and production subsidies and contractual obligations to buy substantial amounts of vaccines in the future. The magnitude and effectiveness of these corporate subsidies remain unknown because COVAX does not divulge the dollar amounts of subsidies provided, and pharmaceutical companies keep purchasing contracts and vaccine manufacturing costs secret. While COVAX socialised some corporate R&D risk, the benefits of vaccine development have been extensively privatised as several vaccine companies are set to make record profits.^2^

A second form of risk sharing within COVAX was to give participating countries access to ‘the world’s largest and most diverse portfolio of COVID-19 vaccines’,^1^ managed by the Coalition for Epidemic Preparedness Innovations (CEPI), to reduce their risk of ending up without a viable product. However, vaccine candidates that gained early approval from the WHO were either not part of the R&D portfolio (Pfizer, Johnson & Johnson) or were distributed in a first instance largely outside of the COVAX Facility (Moderna, AstraZeneca). Meanwhile, CEPI-supported Novavax and Clover, which have entered into large supply deals with COVAX, are yet to gain WHO approval, a prerequisite for use in COVAX.

Finally, COVAX also promised to share collective risk by allowing member countries to buy vaccines as a group and allocate them according to the scientific and ethical expertise of the WHO’s allocation mechanism.^4^ However, COVAX’s governance structure was skewed to favour the interests of its richest members. COVAX allowed them to strike separate unilateral deals with pharmaceutical companies, enabled vaccine cherry-picking from its portfolio and made the world’s poorest countries reliant on aid.^2 5^ Vaccine procurement outside of COVAX soon undermined the initiative’s vaccine supply as manufacturers prioritised deliveries to the highest bidding clients. By mid-October 2021, COVAX had delivered only 344 million of the 2 billion doses it had originally planned to distribute by the end of 2021.^6^ Thus, in the first eighteen months of the pandemic, COVAX offset corporate risk, but failed to ‘share the rewards’ as originally promised. Its goal of vaccine equity remained elusive amidst what the WHO’s Director General has called ‘vaccine apartheid’.

## Dose sharing

Once it became clear that COVAX’s effort to share vaccines equitably through collective purchasing would falter, COVAX pivoted to become a global hub for vaccine ‘dose sharing’. In December 2020, before vaccine roll-outs started, COVAX argued that ‘some countries have secured sufficient doses to begin ‘sharing’ a portion of those doses rapidly with other[s]’.^7^ COVAX was here referring to the wealthy countries who had outbought it, and who had at times either reserved or received many more doses than their populations were likely to require. Against this situation of ‘vaccine hoarding’, COVAX now presented itself as a multilateral dose sharing system (including a no-fault compensation liability scheme) to attenuate its own supply constraints. It pitted ‘dose sharing’ as a superior and more equitable multilateral alternative to bilateral ('i.e., country-to-country) vaccine donations, so-called ‘vaccine diplomacy’.

Like COVAX’s shared purchasing efforts, its dose sharing scheme also struggled. By October 2021, pledging countries had delivered less than 10% of the 1.35 billion doses pledged.^8^ They often only shared those doses that had become redundant, because of domestic vaccine hesitancy, vaccine safety concerns or because of excess supply towards the end of domestic vaccine roll-outs. Moreover, they did not share exclusively through COVAX, often making bilateral donations as well. Eventually, COVAX even accepted to let donor countries earmark doses given to it, contravening its own dose sharing guidelines.^7^ Finally, the quantities and timing of deliveries of doses have often been unpredictable, making it difficult to plan vaccine roll-out, and in some cases forcing recipient governments to turn down shipments.

Many of these operational difficulties resulted from legal challenges. For example, it took lawyers and diplomats eight months to broker legal agreements with Pfizer and Johnson & Johnson to facilitate redistributing COVID-19 vaccines from European countries to poorer nations via COVAX.^9^ The complexities of dose sharing have also led COVAX to consider a range of other kinds of transactions for getting vaccines where they are needed. This includes donor countries giving COVAX’s poorest members priority in manufacturing queues (‘slot-swapping’). COVAX is also considering taking on a new ‘brokerage’ function by enabling rich countries to lend or resell doses secured through bilateral deals, in anticipation of the emergence of a secondary market for vaccines that may further exacerbate global inequities.^10^

## Burden sharing

In February 2021, ‘burden-sharing’ was proposed as a potential new model for financing ACT-A, including COVAX. Burden sharing was, in theory, a way to move away from relying on voluntary aid and frequently unfulfilled pledges, by tapping into countries’ domestic finances instead. The model sought to establish a fair share to be paid by member countries, a way of ‘splitting the bill’ according to macroeconomic indicators including member countries’ gross domestic product (GDP), GDP per capita and the openness of their economies, as defined by the International Monetary Fund.^11^

Such attempts at determining a fair share of contributions based on economic indicators alone seem highly compartmentalised. Is it appropriate to stipulate that Germany, for example, is paying its fair share for global vaccine equity,^12^ when Germany has bought up available vaccines, refused to share doses early on and blocked the temporary reconsideration of intellectual property rights that govern global vaccine production? Given that ‘all burden-sharing models imply some value choices’,^11^ it is critical to question which values have been buried in the weightings of the burden sharing model (largely determining what constitutes a fair share). If fair share contributions should in fact be considered taxes,^11^ this raises the question how mostly unelected officials who govern COVAX and ACT-A, a public–private partnership (PPP) without a democratic governance structure, can claim to represent the people they mean to tax. Solving these conceptual issues may be required to convince countries to contribute. Eighteen months into the pandemic, out of the 91 member countries asked to contribute their fair share to ACT-A, only five (Germany, Norway, Canada, Kuwait and Saudi Arabia) had paid at least the requested amount.^12^

## What is not shared: knowledge, technology and power

COVAX’s raison d’être—global vaccine sharing—has enabled it to posit itself as ‘the only global solution to ending the acute phase of the pandemic’, foreclosing other policy alternatives even as the initiative falters. COVAX has focused its efforts on sharing risk, vaccine doses and money, but it is also defined by what it has *not* attempted to share. This includes the knowledge, intellectual property rights and technology required to produce vaccines at a larger scale and across the globe, and crucially decision-making power.

COVAX is built on a public–private partnership model that espouses voluntary partnership between governments and corporations as the best way to overcome ‘market failures’ and that embraces the current intellectual property regime as a necessary driver of innovation. Thus, it has allowed pharmaceutical companies to keep vaccine contracts and prices secret, and it has defended their resistance to sharing vaccine technology, in spite of globally limited vaccine supply. COVAX pitted itself against the ‘People’s Vaccine’ movement, which has argued vehemently for a temporary waiver of intellectual property rights on COVID-19 tools and vaccines through the Trade-Related Aspects of Intellectual Property Rights (TRIPS) Council at the World Trade Organization. Although the WHO has been an outspoken supporter of the waiver, the UK, Norway, Switzerland and the European Union (EU) have blocked the effort for over a year. As the EU Ambassador to the African Union put it, “Europe Supports COVAX, not the TRIPS waiver.”^13^

At its heart, despite its emphasis on global cooperation, COVAX has actively avoided sharing power. It has been run by unelected officials that work in the established PPPs Gavi and CEPI, with support and influence from the world’s richest governments and private philanthropy, notably the Bill and Melinda Gates Foundation. It fosters their power by bringing multilateral institutions like UNICEF and the WHO into a semiprivatised ‘super-PPP’ structure, and it provides private corporations with direct access to global health policy decision-making.^5^ Donated funds are mostly funnelled to Gavi and CEPI. While South Africa (together with Norway) co-chairs ACT-A’s governing Facilitation Council, further representation from the Global South or the world’s poorest countries is limited in its governance structure.^5^ The publication of a strategic review of ACT-A in October 2021 sparked outrage because only a handful of African experts were consulted, despite Africa’s reliance on COVAX for vaccine access.^14^

## Conclusion: The many meanings of sharing

In the anthropological literature, the term ‘sharing’ denotes a form of exchange that is distinct from other kinds of transactions, such as buying, selling or gift giving. Sharing can be defined as ‘allowing others to access what is positively valued and exposing oneself with others to what is negatively valued’.^15^ It presupposes a reasonably flat power hierarchy between those who are asked to (or choose to) share and those who request it. Sharing also establishes mostly weak and diffuse obligations on the side of those who are given access to a resource, and it can be driven by self-interest as much as by altruistic or other motivations.^15^

COVAX’s strength and importance stem from its early recognition that sharing, as one form of collaboration, is crucial in times of crisis. Whether on a local, national or multilateral scale, sharing resources remains essential for dealing with disasters of all kinds. Yet COVAX has also shown how difficult it is to build multilateral institutions that enable sharing. In a steeply hierarchical setting, powerful groups cannot easily be pressured into sharing. They prefer the clear tit-for-tat logics that mark buying and selling or bilateral vaccine donations. Thus, high-income countries have been able to shirk the responsibility of taking on the health risk of citizens in poorer countries. Pharmaceutical companies have even been able to turn that request onto its head and socialise some of their vaccine production—and sales risks. High-income countries, meanwhile, have shared corporate risk to help develop safe and effective vaccines at record speed. However, they have largely held up sharing these vaccines until they had satisfied their own needs, even for booster doses, often branding their pledges to‘share’ as ‘donations’. In fact, the tendency to conflate terms like ‘sharing’, ‘donating’, ‘transferring’, ‘swapping’ and ‘redistributing’ obscures what COVAX actually *does*. Debates on ‘burden sharing’ have taken no regard of countries’ previous failures to share vaccines, which are arguably relevant for establishing what would constitute a fair share.

## Data Availability

All data relevant to the study are included in the article.
